# Subthalamic Nucleus Stimulation Does Not Have Any Acute Effects on Verbal Fluency or on Speed of Word Generation in Parkinson's Disease

**DOI:** 10.1155/2019/6569874

**Published:** 2019-10-03

**Authors:** Cyril Atkinson-Clement, Friederike Leimbach, Marjan Jahanshahi

**Affiliations:** ^1^Brain and Spine Institute (ICM), Movement Investigation and Therapeutics Team, Paris, France; ^2^Department of Clinical and Movement Neurosciences, UCL Queen Square Institute of Neurology, National Hospital for Neurology & Neurosurgery, London, UK; ^3^The Clinical Hospital of Chengdu Brain Science Institute, MOE Key Lab for Neuroinformation, University of Electronic Science and Technology of China, Chengdu, China

## Abstract

**Background:**

Deep brain stimulation of the subthalamic nucleus (STN-DBS) has been shown to be generally safe from a cognitive perspective, with consistent evidence that the major impact of STN-DBS in Parkinson's disease (PD) is on verbal fluency.

**Objective:**

The aim of this study was first to identify the influence of acute manipulation of STN-DBS in PD on the number and time pattern of word generation on different verbal fluency (VF) tasks, phonemic, switching, and cued switching, and second to determine whether cueing improved VF and if cueing effects interacted with STN-DBS effects.

**Methods:**

Parallel versions of these three verbal fluency tasks were completed by 31 patients with Parkinson's disease who had had bilateral DBS of the STN, twice, with DBS *On* and *Off*, with the order counterbalanced across patients.

**Results:**

There was no effect of acute STN-DBS on the total number of words generated during verbal fluency. As expected, the number of words generated significantly declined over the six 10-second intervals of the verbal fluency tasks, but this time pattern of word generation was not altered by STN-DBS. External cueing significantly increased the number of words generated relative to an uncued switching verbal fluency task, but the cueing effect on VF was not altered by STN-DBS.

**Conclusion:**

In conclusion, (i) acute STN-DBS manipulation did not alter either verbal fluency performance or the time pattern of word generation and (ii) external cueing significantly improved verbal fluency performance both with STN-DBS *On* and *Off*.

## 1. Introduction

Deep brain stimulation (DBS) of the subthalamic nucleus (STN) has been established as an effective treatment of the motor symptoms of Parkinson's disease (PD) (e.g., [1]). STN-DBS has been shown to be generally safe from a cognitive perspective and does not alter major cognitive domains [2, 3]. The results of meta-analyses indicate that the major impact of STN-DBS in PD is on verbal fluency (VF) (e.g., [2, 3]), which is impaired in PD even prior to surgery [4, 5]. The literature on this topic largely performed comparisons of cognitive assessments for PD patients pre- and postsurgery, with only few studies including acute *On* and *Off* STN stimulation comparisons (e.g., [6–9]).

VF is a commonly used assessment in clinical neuropsychological practice, easy to perform but complex to interpret as several executive processes are involved. The completion of VF involves accessing and self-generated search of the lexicon requiring a strategy for organizing and clustering the output, the ability to switch between exemplars (cognitive flexibility), self-monitoring and working memory (to avoid repetitions), self-initiation of responses, and inhibition of inappropriate responses. While previous literature suggests that the postsurgical decline in VF in PD is largely a surgical effect and not a stimulation effect (e.g., [8]), nevertheless the results of the studies which have examined the effect of acute STN stimulation on VF in PD are inconsistent, with some studies reporting detrimental effects of high frequency DBS [10–12], others improvement of switching during verbal fluency [13], and the majority of studies finding no such effects [7–9, 14–16]. However, two of the three studies that reported an acute effect of STN-DBS *On* versus *Off* manipulation simply examined phonemic VF and not other VF tasks. The aim of the present study was first to examine the effect of acute STN stimulation on different VF tasks, phonemic, alternating/switching, and cued switching, and second to determine if STN-DBS alters the time pattern of word generation over the 60 s period of each VF task, and third to determine how external cueing affects VF performance and if stimulation effects interact with provision of external cues for task performance.

## 2. Materials and Methods

### 2.1. Participants

Thirty-one PD patients fulfilling the UK Parkinson's Disease Brain Bank Criteria who had had bilateral STN-DBS were recruited from the Unit of Functional Neurosurgery at the National Hospital for Neurology and Neurosurgery, Queen Square, London. All patients had had STN-DBS for at least 6 months. Prior to surgery all patients were screened and had a detailed neuropsychological assessment to confirm absence of major cognitive impairment and major psychiatric disorder. At the time of assessment, specific aspects of cognitive function were assessed (trail making test and stroop color-word interference test), as well as mood (Hospital Anxiety and Depression Scale (HADS)), apathy (Starkstein Apathy Scale), perceived fatigue (Likert scale for physical and mental fatigue), and motor functions (Unified Parkinson's Disease Rating Scale part-III [UPDRS]). Patients were assessed twice on the same day and under current medication, once with STN-DBS activated (*On*) and once with stimulation switched off (*Off*), with the order counterbalanced across patients and on parallel versions of the verbal fluency tasks. The DBS was turned off for half an hour before testing in the off DBS state was started. This period is considered sufficient for the effects of stimulation to dissipate and has been commonly used in DBS on vs off studies. This project was approved by the joint Ethics committee of the UCL Institute of Neurology and the National Hospital for Neurology & Neurosurgery and all patients gave informed consent. Demographic and clinical information and STN-DBS parameters are provided in Table 1.

### 2.2. Tasks

The protocol included three verbal fluency tasks composed of three subtasks performed in a random order: one phonemic task (letters F, A, and S (FAS)); switching task (switching between two semantic categories, switching between two letters, and switching between a letter and a semantic category; with the former two tasks requiring intradimensional switching while the latter task involved extradimensional switching); and a cued switching task (identical to the switching task but with provision of external cues providing participant about the nature of the task on each trial). For each subtest, patients had one minute to generate words meeting the task criteria. Patients were instructed to generate words meeting the criteria as fast as possible, to avoid proper nouns and numbers. For each test, the total number of words generated was calculated, discarding repetitions and intrusions and words not meeting the criteria. The output of the patients for each of the VF tasks was tape-recorded, and the number of words generated in each 10-second epoch of the 60 seconds period was documented. In addition, the cluster size (number of words for a same category or letter minus one) and the number of switches (number of transitions between clusters) were calculated as previously described [17]. Parallel versions of the verbal fluency tasks were used for the DBS on versus off assessments.

### 2.3. Statistical Analyses

All statistical analyses were conducted with the R software, and the statistical threshold was set as *p* < 0.05. Clinical and demographic comparisons consisted of the Kruskal–Wallis test. For VF tasks, we ran repeated measures analysis of variance including systematically both the effects of STN-DBS (*On* and *Off*) and task (FAS phonemic, switching, and cued switching). The time effect, the number of words generated for each of the six 10-second intervals, was also derived. We considered 6 different variables: total number of correct words generated, number of errors (intrusions and repetitions), phonemic and semantic clusters size, and number of phonemic and semantic switches. A Bonferroni correction was performed. Lastly, multiple linear regressions were performed between the previous 6 variables and the trail making test and Stroop test results.

## 3. Results

The mean and standard deviation of the measures of cognition, mood, and behaviour are presented in Table 1. We found no differences *On* versus *Off* STN-DBS on any of the measures of cognition or mood (all *p* > 0.05) except for physical fatigue (*p*=0.03) which was higher with DBS *Off* and the severity of motor symptoms which was improved with DBS *On* (*p* < 0.0001), which indirectly confirms the correct localization of at least one electrode contact in the motor STN.

**Figure 1:**
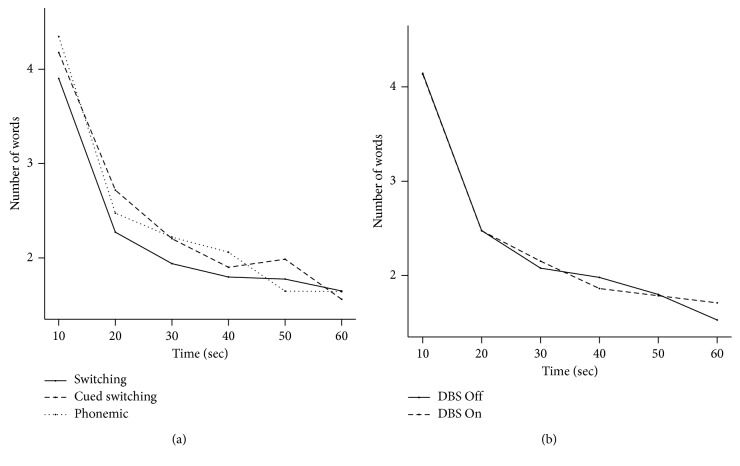
The mean number of words generated during the 10-second intervals of the three verbal fluency tasks (a) and with STN-DBS *On* or *Off* (b).

**Figure 2:**
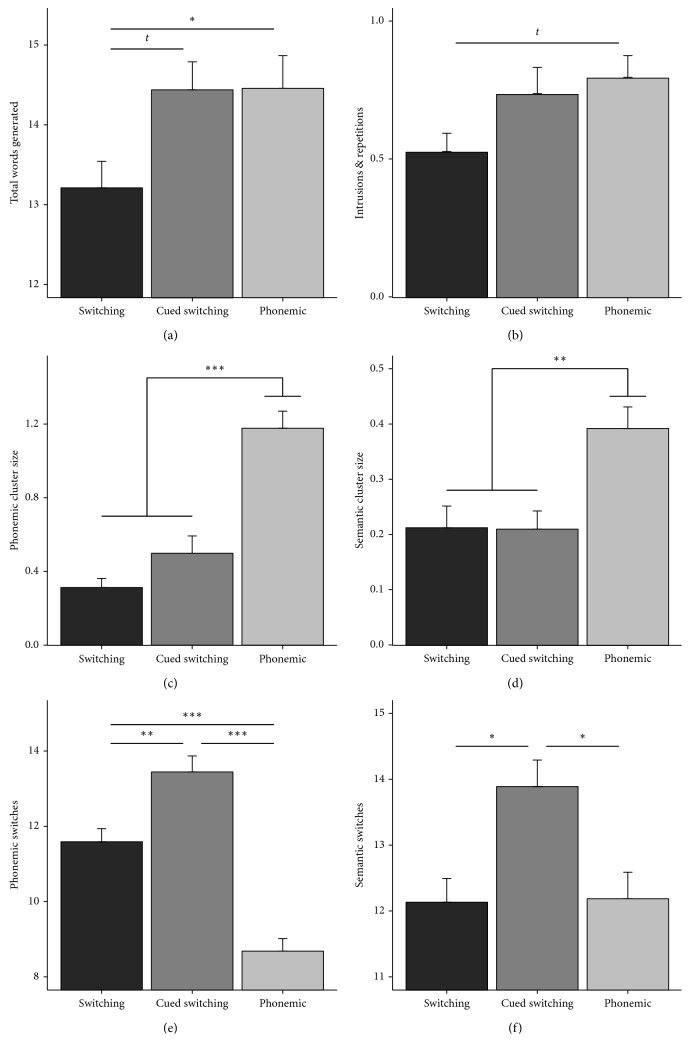
The average number of words generated (a), intrusions and repetitions (b), the number of phonemic cluster size (c), semantic cluster size (d), phonemic switches (e), and semantic switches (f) for the three verbal fluency tasks. *t*: *p* < 0.1; ^*∗*^: *p* < 0.05; ^*∗∗*^: *p* < 0.1; ^*∗∗∗*^: *p* < 0.001.

The mean and standard errors for the number of words generated on each of the three VF tasks for each 10-second periods and for the two STN-DBS conditions are presented in Figure 1. The main effect of STN-DBS and the interactions of STN-DBS condition with the nature of VF task or generation time period were not significant (all *p* > 0.05). As a result, Figure 2 shows the number phonemic and semantic cluster size and switches for each of the three VF tasks averaged across the two STN-DBS conditions.

As expected, for all three VF tasks, we observed a decrease in the number of words generated across the six 10-second periods (*F*_(5;3032)_ = 389.3; *p* < 0.0001). There was also a significant effect of the nature of the VF task (*F*_(2;3032)_ = 8.78; *p* < 0.0001), as well as a significant interaction between VF tasks and generation period (*F*_(10;3032)_ = 3.06; *p*=0.0007). Regarding the interaction between VF tasks and generation period, post hoc analyses highlighted that the number of generated words during the switching task was below the number of generated words during the two other tasks, especially during the first 40 seconds (lower than the phonemic task at 10 seconds (*p*=0.0003), 30 seconds (*p*=0.03), and 40 seconds (*p*=0.04) and lower than the cued switching task at 20 seconds (*p*=0.002)). These results were not influenced by handedness, which did not influence the number of words generated (*F*_(1;2997)_ = 0.253; *p*=0.62).

We found a significant influence of the VF task on the total number of words generated (*F*_(2;497)_ = 6.92; *p*=0.001), on total errors (*F*_(2;522)_ = 3.69; *p*=0.025), and on phonemic and semantic cluster size (*F*_(2;436)_ = 34.5, *p* < 0.0001; *F*_(2;439)_ = 18.1, *p* < 0.0001, respectively) and phonemic and semantic switching (*F*_(2;436)_ = 56.9, *p* < 0.0001; *F*_(2;439)_ = 4.62, *p*=0.01, respectively; Figure 2). Post hoc tests showed that patients generated significantly more words during the FAS phonemic VF and the cued switching tasks than the switching task (*p*=0.068 and *p*=0.05, respectively), and they made significantly more errors on the phonemic VF than the switching task (*p*=0.058). The sizes of phonemic and semantic clusters were significantly higher for the phonemic VF task than for the switching (*p* < 0.0001 and *p*=0.003, respectively) and the cued switching (*p* < 0.0001 and *p*=0.006, respectively) tasks. By contrast, the number of phonemic and semantic switches were significantly higher in the cued switching task than in the phonemic VF (*p* < 0.0001 and *p*=0.017, respectively) and the switching (*p*=0.005 and *p*=0.016, respectively) tasks. None of these effects were influenced by the patients' handedness (*p* > 0.05).

The trail making test (time difference between parts B and A) was associated with the total number of words generated for the VF switching (slope = −9.25; *p*=0.002) and cued switching tasks (slope = −9.32; *p*=0.004). The Stroop (interference time) and the size of semantic clusters for the VF switching (slope = −24.45; *p*=0.004) and cued switching tasks (slope = −32.8; *p*=0.016) showed significant associations as well.

## 4. Discussion

Our data revealed two main findings. First, VF performance and time course of word generation pattern were not altered by acute manipulation of STN-DBS. Second, external cueing significantly improved VF performance, but this was not altered by STN-DBS.

The first finding of our study relates to the absence of an effect of acute manipulation of STN-DBS *On* vs *Off* on verbal fluency, and especially on the time pattern of word generation. This is consistent with the majority of previous studies which also did not find an acute effect of STN stimulation on VF (e.g., [6–9]). Our results further expand these findings by establishing that the number of words generated across the six 10-second periods of each VF task was not affected by acute STN stimulation, thus showing that STN-DBS does not alter the speed or rate of word generation during VF. Some previous studies examining the surgical effect of STN-DBS on VF have suggested that the postsurgical decline in VF results from “an intact lexicon running slowly” [18] or a slower speed of processing [19]. However, in both these studies, this conclusion was based on correlations between VF tests and independent measures of speed of processing such as a lexical decision task or symbol search and digit symbol coding rather than direct measurement of the number of words generated across time in each VF task as in the present study. Based on the current results, acute STN stimulation does not affect the speed of word generation during VF tasks.

In relation to the impact of STN-DBS on VF, two hypotheses were formulated, corresponding to the direct effect of STN stimulation on VF (e.g., [20]) or a lesional effect due to neurosurgery (e.g., [8, 9, 21]). Some previous studies suggested that STN-DBS induces decline in VF due to the electrode trajectory or position. However, there is no consensus of evidence, with some results suggesting that electrodes in or near the STN proper are associated with VF deficits [22], whereas others have reported electrode trajectories that intersected the caudate nucleus [23, 24] to be critical, whereas still others have failed to find microelectrode recordings or electrode position in the STN (ventral vs dorsal) to influence VF decline [8, 25, 26]. However, since information regarding the electrode coordinates was not available, we cannot address this issue. In a previous study recording local field potentials from the DBS electrodes inserted in the STN bilaterally in PD patients, we found a significant association between increased gamma band activity in the local field potentials and switching during verbal fluency relative to a control word repetition task, suggesting that STN activity is significantly modulated during VF performance [27]. This combined with the results of the studies which have reported a direct effect of stimulation frequency (e.g., [12, 28, 29]) and of surgical procedure on VF (e.g., [21, 30–32]) and our results suggest that VF impairment following STN-DBS cannot be attributed solely to a surgical lesional effect or only to STN stimulation, but to some combination of the two effects, or some other as yet unexplored factors, which needs to be investigated and clarified in future studies. Other issues such as the contribution of postoperative apathy and dysarthria and changes in levodopa medication to VF decline after STN-DBS have been considered, and the evidence relating to these is also contradictory [3, 8, 33–36].

As expected, we observed that external cueing improved switching VF. A previous study in unoperated patients observed that the provision of an external cue improved VF in PD in the sense that it helped to decrease the number of perseverative errors [37]. Similar external cueing effects have been reported for different cognitive processes related to problems solving [38] freezing of gait [39], timing of simple finger lifting movements [40], and finger tapping [41]. Together, these results contribute to the hypothesis that PD patients have problems with self-generation of actions and solutions. Regarding the neural processes, self-initiated movements were associated with increased activation of the dorsolateral prefrontal cortex relative to externally cued movements [40] and an increased activation of the cerebellum, probably to compensate for the basal ganglia dysfunction [41], which could partly explain why PD patients in our study were helped by cueing.

VF performance has been considered to involve two main cognitive processes: clustering and switching. Previous studies have suggested that switching during VF is dependent on frontal executive functions, whereas clustering reflects activation of the temporal cortical networks [42]. As it is evident from Figure 2, the phonemic, switching, and cued switching tasks differed in terms of cluster size and number of switches. These results are consistent with previous findings [43]. We also observed an association between the VF switching tasks (with and without cues) and the trail making test and the Stroop test, which involves cognitive flexibility and set-switching and inhibition. The results of the imaging studies which have examined the neural substrates of STN-DBS induced VF impairments in PD are inconsistent [11, 36, 44]. Our findings regarding the differences between VF tasks suggest that the heterogeneity of results across studies may partly be due to the specific nature of the verbal fluency tasks used.

## 5. Conclusions

In conclusion, our results indicate that (i) the VF impairment following STN-DBS is not due to an acute STN stimulation effect and acute stimulation does not alter the number of words generated and their time pattern across the 60 seconds interval and (ii) external cueing improved VF performance in PD, but this was not influenced by STN stimulation either.

## Figures and Tables

**Table 1:** Demographic and clinical characteristics of the Parkinson's disease patients, and the cognitive and mood measures *Off* and *On* STN-DBS.

	*Off*	*On*	*p* value
*Demographic*			
Number of participants (F/M)	31 (10/21)	—	—
Years of education	14.1 (3.2)	—	—
Age	58.8 (6.9)	—	—
Handedness (L/R)	5/26	—	—

*Clinical*			
Disease duration (years)	14.7 (4.7)	—	—
UPDRS-III	29.8 (17.1)	11.7 (8.1)	**0.0001**
STN-DBS voltage—L	—	2.7 (0.8)	—
STN-DBS voltage—R	—	2.9 (0.7)	—
STN-DBS frequency—L	—	136 (13.8)	—
STN-DBS frequency—R	—	133.3 (9.9)	—
STN-DBS pulse—L	—	62.13 (7.7)	—
STN-DBS pulse—R	—	61.10 (5.6)	—

*Cognition*			
Trail making test (time—part A)	47.3 (20.6)	41.2 (27.5)	0.06
Trail making test (time—part B)	120.1 (107.9)	115.1 (105.9)	1
Trail making test (time difference parts B and A)	72.8 (92.7)	72.8 (80.6)	0.51
Stroop (interference, time)	69.2 (50.5)	67.4 (59.9)	0.42
Stroop (interference, errors)	2.5 (3)	2.4 (3.7)	0.78

*Mood and behaviour*			
HADS—anxiety	5.8 (3.4)	5.2 (3)	0.54
HADS—depression	4.9 (2.4)	5.1 (2.9)	0.92
Apathy	10.5 (5.5)	12.6 (7.9)	0.32

*Fatigue*			
Physical	6.1 (3.2)	4.1 (2.9)	**0.03**
Mental	6.4 (2.5)	5.1 (2.3)	0.10

Results are given as mean ± SD (Kruskal–Wallis test). F: female; HADS: Hospital Anxiety and Depression Scale; L: left; M: male; PD: Parkinson's disease; R: right; STN-DBS: Subthalamic deep brain stimulation; UPDRS: Unified Parkinson's Disease Rating Scale.

## Data Availability

The data underlying this study are available from the corresponding author upon request and excluding commercial activity.
